# Symptoms of School Burnout in Comprehensive School and Mental Health Problems in Late Adolescence

**DOI:** 10.1111/sjop.70064

**Published:** 2025-12-26

**Authors:** Milja Parviainen, Kaisa Aunola, Minna Torppa, Karoliina Koskenvuo, Miia Saarikallio‐Torp, Anna‐Maija Poikkeus, Kati Vasalampi

**Affiliations:** ^1^ Department of Psychology, Faculty of Education and Psychology University of Jyväskylä Jyväskylä Finland; ^2^ Department of Teacher Education, Faculty of Education and Psychology University of Jyväskylä Jyväskylä Finland; ^3^ The Social Insurance Institution of Finland Kela Finland; ^4^ Department of Education, Faculty of Education and Psychology University of Jyväskylä Jyväskylä Finland

**Keywords:** antidepressants, cynicism, depressive symptoms, exhaustion, mental health problems, symptoms of school burnout

## Abstract

The aim of the present study was to examine the extent to which symptoms of school burnout (exhaustion and cynicism) during the comprehensive school years played a role in individuals' long‐term mental health problems. The data were collected in four municipalities (i.e., one big, one rural, and two medium‐sized) around Finland. The sample included 673 students (376 girls, 297 boys) whose symptoms of school burnout were assessed three times: in Grade 6 (ages 12–13), Grade 7, and Grade 9. Indicators of later mental health problems were the use of antidepressants at the ages of 16–20 years old and self‐reported depressive symptoms during the first year of upper secondary education at ages of 16–17 years. Information on symptoms of school burnout and depressive symptoms was derived from self‐reports, and information on the use of antidepressants was obtained from the National Drug Prescription Register data. The data were analyzed using logistic regression and hierarchical linear regression models. The results showed that symptoms of exhaustion in Grade 9 significantly predicted the use of antidepressants in late adolescence; the higher the level of exhaustion in Grade 9, the greater the likelihood of using antidepressants during the follow‐up period. Similar findings were found for self‐reported depressive symptoms, as higher levels of exhaustion in Grades 7 and 9 predicted significantly higher levels of subsequent self‐reported depressive symptoms. Higher levels of cynicism in Grade 9 predicted significantly higher levels of subsequent self‐reported depressive symptoms, but not the use of antidepressants. The findings indicated that school burnout symptoms are significant predictors of ongoing mental health problems, emphasizing the need for early preventive work and interventions. Symptoms of exhaustion and cynicism increase the risk of depressive symptoms, and exhaustion may also progress to clinical depression requiring medication.

## Keypoints

- Symptoms of exhaustion in Grade 9 significantly predicted later use of antidepressants and higher self‐reported depressive symptoms in upper secondary school.
- Symptoms of cynicism in Grade 9 predicted increased self‐reported depressive symptoms, but it did not predict antidepressant use.
- School burnout symptoms are significant predictors of later mental health problems, emphasizing the need for early preventive work and interventions.

## 1 Introduction

Although a majority of students cope well with study‐related pressures, many students experience that their studies are too demanding and show symptoms of school burnout (Finnish Institute for Health and Welfare 2024). The school burnout phenomenon is worryingly widespread, and students in primary/middle school are already experiencing symptoms of school burnout (Parviainen et al. 2021; Salmela‐Aro et al. 2016; Tomaszek et al. 2024). Moreover, symptoms of school burnout may increase over time as students move forward on their educational paths (Lee and Lee 2018). In particular, upper secondary education students on an academic track are at risk of developing symptoms of school burnout (Salmela‐Aro and Tynkkynen 2012; Söderholm et al. 2025).

The concept of “burnout” in the sense of a human condition was first examined in a work context (Maslach et al. 2001), but the concept has also been applied more broadly, including in educational settings (for a review, see Vansoeterstede et al. 2023). According to a widely used definition (Salmela‐Aro, Kiuru, et al. 2009), school burnout consists of three dimensions: exhaustion, cynicism, and a sense of inadequacy. Exhaustion refers to experiences of school‐related strain that bothers students also during their spare time, and students experiencing exhaustion, for example, can sleep poorly because of concerns regarding their schoolwork. Cynicism is manifested as a lack of interest and motivation in schoolwork, and students experiencing cynicism ponder whether schoolwork has any meaning. A sense of inadequacy refers to students' school‐related feelings of incompetence. According to the study demands‐resources model (Salmela‐Aro et al. 2022; Salmela‐Aro and Upadyaya 2014; see also Bakker et al. 2003), school burnout occurs when the demands (e.g., academic pressure and challenging tasks) faced by a student exceed the available resources (e.g., social support and motivational resources).

Unlike depression, burnout has not been classified as a medical condition (World Health Organization, n.d.), and researchers of work burnout have debated whether it and depression overlap or are separate constructs (Bianchi et al. 2021; Koutsimani et al. 2019). Similarly, the symptoms of depression and those of school burnout have been shown to be closely linked in several studies (Fiorilli et al. 2017; Olivier et al. 2023; Salmela‐Aro, Savolainen, and Holopainen 2009), but the current view is that while depressive symptoms can be more pervasive in their nature and origin, symptoms of school burnout are specific to the school context and result from prolonged and chronic school‐related stress (Salmela‐Aro, Kiuru, et al. 2009).

Previous research has established a link between school burnout and indicators of psychological distress, such as higher academic stress, internalizing symptoms, poorer self‐esteem, and lower levels of life satisfaction (Jiang et al. 2021; Parviainen et al. 2021; Salmela‐Aro and Upadyaya 2014). However, it remains unclear whether school burnout symptoms serve as long‐term predictors of clinically significant mental health conditions. To address the limitations of previous research, the present study was designed to examine the extent to which symptoms of school burnout during comprehensive school years are linked to continuing later mental health problems. The use of antidepressants was chosen as a primary indicator of mental health problems in late adolescence because the use of antidepressants can be considered an indicator of clinically significant mental health problems (e.g., depression and anxiety) because an authorized physician has evaluated an individual's condition and determined it required pharmaceutical treatment. Information regarding the use of antidepressants was obtained from the National Drug Prescription Register data maintained by Kela (The Social Insurance Institution of Finland). Using register data provided not only a more objective indicator of antidepressant use than self‐reports but also helped to avoid the shared‐method problem often present in studies that rely on self‐reports to assess both explanatory and outcome variables.

More specifically, this study examined to what extent symptoms of school burnout (exhaustion and cynicism) in primary school (Grade 6, ages 12–13 years old) and lower secondary school (Grades 7 and 9) were associated with the use of antidepressants after Grade 9 (ages 16–20 years old). The focus was on comprehensive school years because school burnout symptoms begin to emerge relatively early, and our goal was to examine how early they play a role in future mental health issues. Although there were no previous studies found on the predictive role of symptoms of school burnout on the use of antidepressants years later, it was hypothesized that symptoms of school burnout evident during comprehensive school would predict a subsequent higher likelihood of antidepressant usage. This hypothesis was based on previous findings that have connected the symptoms of school burnout to psychological ill‐being (Fiorilli et al. 2017; Jiang et al. 2021; Parviainen et al. 2021) and findings linking job burnout to the use of antidepressants (Leiter et al. 2013; Madsen et al. 2015).

To validate the results regarding the key mental health problem outcome measure (i.e., the use of antidepressants) as an additional research question, we examined whether the symptoms of school burnout during comprehensive school also predicted self‐reported depressive symptoms right after its completion (at an age of 16–17 years old). Based on previous studies (Olivier et al. 2023; Salmela‐Aro, Savolainen, and Holopainen 2009), it was hypothesized that higher levels of symptoms of school burnout would be linked to subsequent higher levels of self‐reported depressive symptoms. In addition, the role of gender was taken into account in the analyses, as previous studies have shown that there are gender differences in patterns of mental health problems. Prior studies have often shown girls to be at a higher risk of experiencing exhaustion (Herrmann et al. 2019) and depressive symptoms (Shorey et al. 2022) than boys.

Overall, we hypothesized that school‐related problems may generalize beyond the school context. Regarding cynicism, the mechanism could be that when a student loses a sense of meaning in school, this lack of meaning spills over to other contexts and leads to a loss of meaning in life more broadly (Wang et al. 2024). When it comes to the connection between exhaustion and depression, it is possible that becoming overwhelmed by schoolwork and sleeping poorly due to school stress may lead to more generalized fatigue, making even simple everyday tasks and activities feel difficult. Exhausted students may also be more likely to withdraw from things that previously brought them joy, such as hobbies and social activities. Symptoms of school burnout and depression have also been linked to sleep problems (Liu et al. 2021; Lovato and Gradisar 2014), which could be one mechanism linking them.

## 2 Materials and Methods

### 2.1 Participants and Procedure

The data used in the present study were from the First Steps follow‐up study (Lerkkanen et al. 2006–2016) and its extension, that is, the School Path: From the First Steps to Secondary and Higher Education study (Vasalampi and Aunola 2016–2023). In the former study, approximately 2000 participants born in 2000 in four Finnish municipalities (i.e., one big, one rural, and two medium‐sized) were followed from kindergarten to lower secondary school (2006–2016). In the extension, the participants took part in the study twice during their upper secondary education studies (2017–2019). The present study used questionnaire data that were collected at four different time points (T1–T4) over the course of 5 years (2013–2017). Each data collection took place in the spring semester. The data included the following time points: Grade 6 in the final year of primary school (T1, 12–13 years of age, April 2013); Grade 7 in the first year of lower secondary school (T2, 13–14 years of age, April 2014); Grade 9 in the final year of lower secondary school (T3, 15–16 years of age, April 2016); and first year of the upper secondary education (T4, 16–17 years of age, January through March 2017). Questionnaire data were collected during normal school lessons by trained researcher assistants at T1–T3 and trained research assistants or teachers at T4. Parents/guardians (T1–T3) or participants (T4) provided written informed consent for participation in the study.

In addition to longitudinal questionnaire data, information on antidepressant medication use was retrieved from the National Drug Prescription Register data maintained by Kela (The Social Insurance Institution of Finland). The register contains information on all reimbursed outpatient prescription medicine purchases in Finland. The Anatomical Therapeutic Chemical (ATC) classification system was used (for more information, see WHO Collaborating Centre for Drug Statistics Methodology 2025). Register data from January 1999 through December 2020 were included, covering the medication reimbursement history of the participants from birth (a few participants were born in 1999 but most in 2000). Permission for register linkage was obtained from the participants in upper secondary education (semester 2018–2019).

The final data comprised 673 students (55.9% girls) who gave permission for register linkage and had participated in the study at Grades 6, 7, or 9 and had not used antidepressants in comprehensive school. Exclusion of participants who had used antidepressants during comprehensive school (*n* = 18) was done to examine whether symptoms of school burnout were linked to subsequent antidepressant usage instead of reflecting continuation of use. In other words, the goal was to predict the initiation of medication use rather than its continuation. Specifically, we examined whether early school burnout symptoms could predict if a person would later need medical treatment. The numbers of students who participated in the study by education level were as follows: Grade 6, 626 students (mean age = 12.752, SD = 0.305); Grade 7, 632 students (mean age = 13.750, SD = 0.302); Grade 9, 636 students (mean age = 15.731, SD = 0.309); and upper secondary education, 619 students (mean age = 16.676, SD = 0.346).

Ethical statements for the follow‐up study were obtained in 2006 and 2018 from the Ethical Committee of the University of Jyväskylä.

### 2.2 Educational System in Finland

In Finland, children start pre‐primary education in August of the year in which they turn 6 years old. A year later, they enter the 9‐year comprehensive school, which consists of primary school (Grades 1–6) and lower secondary school (Grades 7–9). After comprehensive school, students apply for upper secondary education, typically either a general upper secondary school (i.e., academic track) or a vocational school (i.e., vocational track), which typically takes 3 years to finish. After upper secondary education, students can apply for higher education (i.e., a university or university of applied sciences).

### 2.3 Measures

#### 2.3.1 Symptoms of School Burnout

The School Burnout Inventory (SBI; Salmela‐Aro, Kiuru, et al. 2009) was used to assess symptoms of school burnout using two of the subscales: exhaustion and cynicism. Exhaustion was measured with three items (“I often sleep badly because of matters related to my schoolwork”; “I brood over matters related to my schoolwork a lot during my free time”; and “The pressure of my schoolwork disturbs my life outside school”) and cynicism likewise with three items (“I feel a lack of motivation in my schoolwork”; “I feel that I am losing interest in my schoolwork”; and “I'm continually wondering whether my schoolwork has any meaning”). The items were rated on a 5‐point Likert scale (1 = “completely disagree”; 5 = “completely agree”). A composite score was calculated separately for exhaustion and cynicism for the three measurement points (T1, T2, and T3) as the mean of the items measuring the constructs. The Cronbach's alpha reliability coefficients for exhaustion were 0.831, 0.841, and 0.857 for T1, T2, and T3, respectively, and for cynicism were 0.815, 0.831, and 0.854, respectively.

#### 2.3.2 Use of Antidepressants

Information about antidepressants purchased for the participants was obtained from the National Drug Prescription Register data maintained by Kela (The Social Insurance Institution of Finland) using ATC codes from category N06A. Based on the dispensing date of the prescriptions, participants were divided into two categories: 0 = no antidepressant purchases at all (*n* = 594, 88.3%); 1 = two or more purchases after comprehensive school (i.e., January 1, 2017, and beyond) but no purchases before that (*n* = 64, 9.5%). Those with only one purchase (*n* = 15, 2.2%) were excluded from the analyses regarding the use of antidepressants. The rationale was that only after more than one purchase was an individual considered to be using antidepressants systematically for treatment.

#### 2.3.3 Self‐Reported Depressive Symptoms

Depressive symptoms at upper secondary education (T4) were assessed with the Depression Scale (DEPS; Salokangas et al. 1995). Ten items focusing on depressive symptoms during the previous month (e.g., “I felt blue” and “I did not enjoy my life”) were rated on a 4‐point Likert scale (1 = “not at all”; 4 = “very much”). A composite score was calculated for depressive symptoms as the mean of the items measuring the construct. The Cronbach's alpha reliability coefficient for depressive symptoms was 0.930.

### 2.4 Data Analysis

The data were analyzed using logistic regression models, as the outcome variable (i.e., use of antidepressants) included two categories: 0 = no purchases or 1 = at least two purchases. The first step in the data analysis was to examine whether there were interactions between gender and the school burnout variables (T1–T3, i.e., Grades 6, 7, and 9) when predicting the use of antidepressants after comprehensive schooling. These analyses were conducted separately for exhaustion and cynicism at each time point T1–T3. In the analyses, gender and exhaustion or cynicism variables at a particular time point, as well as the interaction term of gender X exhaustion or cynicism, were included as independent variables. The interactions between gender and the school burnout variables were all statistically non‐significant, indicating that gender did not moderate any of the regression paths. Therefore, the interaction terms were not included in the final models. In the final model, the effect of gender was controlled for by including gender in the first step of the model as an independent variable. Gender was used in the analysis as a categorical variable (0 = girl, 1 = boy). In the second step, the school burnout variables (exhaustion and cynicism) from the three time points T1–T3 (i.e., Grades 6, 7, and 9) were added to the model.

In order to validate the outcome measure (the use of antidepressants), we next examined further whether the symptoms of school burnout during comprehensive school (T1–T3) predicted self‐reported depressive symptoms after the completion of comprehensive schooling (T4, 16–17 years old). This was examined using a hierarchical linear regression model in which gender was included in the first step as a covariate and the school burnout variables (exhaustion and cynicism) from the three time points T1–T3 were included as independent variables in the second step.

## 3 Results

The descriptive statistics (means, standard deviations, and intercorrelations) of the study variables are provided in Table 1.

**TABLE 1:** Descriptive information and correlations between variables included in the study.

	1	2	3	4	5	6	7	8	9
Exhaustion T1									
2 Exhaustion T2	0.55***								
3 Exhaustion T3	0.41***	0.47***							
4 Cynicism T1	0.41***	0.18***	0.09*						
5 Cynicism T2	0.31***	0.37***	0.13**	0.54***					
6 Cynicism T3	0.21***	0.19***	0.38***	0.39***	0.48***				
7 Depressive symptoms T4	0.25**	0.26***	0.37***	0.13**	0.13**	0.34***			
8 Use of antidepressants^a^	0.09*	0.06	0.19***	0.04	0.03	0.15***	0.35***		
9 Gender^b^	−0.05	−0.05	−0.26***	0.09*	0.15***	0.08	−0.21***	−0.12**	
*N*	626	632	636	626	632	636	619	658	673
Mean	2.00	2.09	2.38	2.16	2.09	2.15	1.50	0.10	0.44
Standard deviation	0.92	0.94	1.04	0.92	0.87	0.96	0.58	0.30	0.50
Minimum, maximum	1, 5	1, 5	1, 5	1, 5	1, 5	1, 5	1, 4	0, 1	0, 1

Abbreviations: T1, Time 1 (Grade 6); T2, Time 2 (Grade 7); T3, Time 3 (Grade 9); T4, Time 4 (the first year of upper secondary education).

* *p* < 0.01.

** *p* < 0.01.

*** *p* < 0.001.

^a^ 0, no antidepressants; 1, antidepressants between age 16 and 20.

^b^ 0, girl; 1, boy.

Firstly, we hypothesized that symptoms of school burnout evident during comprehensive school would predict a subsequently higher likelihood of antidepressant usage. We thus examined whether cynicism and exhaustion at Grades 6, 7, and 9 (i.e., T1–T3, respectively) were associated with the use of antidepressants at the ages of 16–20, after controlling for the effect of gender. The results of the logistic regression model (*ꭓ* ^2^(7) = 27.893, *p* < 0.001) are shown in Table 2. According to Nagelkerke's *R* ^2^ value, gender as a covariate explained 2.1% of the variance in the use of antidepressants before entering the school burnout variables into the model. After the addition of the school burnout variables, the amount of explained variance increased to 10.4%, and the effect of gender was no longer statistically significant. The results (see Table 2) showed that exhaustion in Grade 9 statistically significantly (*p* = 0.008) predicted the use of antidepressants; the higher the level of exhaustion in Grade 9, the greater the likelihood of using antidepressants during the follow‐up period.

**TABLE 2:** Logistic regression analysis results—symptoms of school burnout at grades 6 (time 1), 7 (time 2), and 9 (time 3) as predictors of the use of antidepressants (0 = no use of antidepressants, 1 = use of antidepressants) at Age 16*–*20.

	*β*	s.e.	sig.	OR	(95% CI)
STEP 1					
Gender	−0.722	0.317	0.023	0.486	(0.261*–*0.904)
STEP 2					
Gender	−0.536	0.345	0.120	0.585	(0.297*–*1.151)
Exhaustion/grade 6	0.081	0.206	0.694	1.084	(0.724*–*1.624)
Cynicism/grade 6	−0.077	0.207	0.709	0.926	(0.617*–*1.388)
Exhaustion/grade 7	−0.150	0.201	0.458	0.861	(0.580*–*1.278)
Cynicism/grade 7	0.066	0.224	0.768	1.068	(0.689*–*1.657)
Exhaustion/grade 9	0.464	0.176	0.008	1.591	(1.127*–*2.247)
Cynicism/grade 9	0.337	0.183	0.065	1.401	(0.979*–*2.004)

*Note:* The independent variables in the model explained 10.4% of the variation in the use of antidepressants (Nagelkerke's *R* ^2^). From independent variables, statistically significant effect was found for exhaustion in Grade 9: the higher the level of exhaustion in Grade 9, the greater the likelihood of using antidepressants.

Abbreviations: 0, girl; 1, boy; 95% CI, 95% confidence interval; OR, odds ratio; s.e., standard error; *β*, standardized beta coefficient.

Secondly, we hypothesized that higher levels of symptoms of school burnout during comprehensive school would be linked to subsequent higher levels of self‐reported depressive symptoms. Therefore, we next examined whether students' symptoms of school burnout in comprehensive school (T1–T3) predicted subsequent self‐reported depressive symptoms after the completion of comprehensive schooling (T4, 16–17 years old), after controlling for the effect of gender. The results of the hierarchical linear regression model (*F*(7, 512) = 24.005, *p* < 0.001) are presented in Table 3. At the first step, gender was included in the model. Gender predicted 4.8% (*p* < 0.001) of the self‐reported depressive symptoms, with girls reporting higher levels than boys. At the second step, school burnout variables were added to the model, and the prediction of depressive symptoms increased to 24.7% (Δ*R* ^2^ = 0.199, *p* < 0.001). The results showed that higher levels of exhaustion in Grades 7 (*p* < 0.05) and 9 (*p* < 0.01) predicted statistically significant higher levels of subsequent self‐reported depressive symptoms. In addition, higher levels of cynicism in Grade 9 (*p* < 0.001) predicted statistically significant higher levels of subsequent self‐reported depressive symptoms.

**TABLE 3:** Hierarchical Regression Analysis Results—School Burnout Symptoms at Grades 6 (Time 1), 7 (Time 2), and 9 (Time 3) as Predictors of Self‐Reported Depressive Symptoms During the First Year of Upper Secondary Education (Time 4).

	STEP 1	STEP 2
	*β*	*β*
Gender	−0.220***	−0.179***
Exhaustion/grade 6		0.080
Cynicism/grade 6		0.023
Exhaustion/grade 7		0.121*
Cynicism/grade 7		−0.065
Exhaustion/grade 9		0.147**
Cynicism/grade 9		0.290***
	*F*(1, 518) = 26.244***	*F*(7, 512) = 24.005***
	*R* ^*2*^_change_ = 0.048***	*R* ^*2*^_change_ = 0.199***

*Note:* The independent variables explained 24.7% of the variation in self‐reported depressive symptoms, the proportion of school burnout variables from this being 19.9%. From school burnout variables, exhaustion in Grades 7 and 9, and cynicism in Grade 9, have a statistically significant positive effect on the explained variance.

Abbreviations: 0, girl; 1, boy; *β*, standardized beta coefficient.

* *p* < 0.05.

** *p* < 0.01.

*** *p* < 0.001.

Overall, the findings indicated that exhaustion in Grade 9 was the strongest predictor of later antidepressant use, while both exhaustion and cynicism predicted self‐reported depressive symptoms. Additional analyses (see Appendix A) that controlled for parental level of education showed that no statistically significant associations emerged between parental education and students' use of antidepressants or between parental education and the self‐reported depressive symptoms. Thus, adding parental education to the tested models did not affect the results reported above (Tables A1 and A2).

## 4 Discussion

Although symptoms of school burnout have often been linked to various indicators of psychological ill‐being (Jiang et al. 2021; Parviainen et al. 2021; Salmela‐Aro and Upadyaya 2014), very little is known about to what extent symptoms of school burnout in comprehensive school are linked to individuals' mental health in the long run. The findings of the present longitudinal study suggest that psychological ill‐being during comprehensive school is linked to continuing mental health problems. The results demonstrated that higher levels of school‐related exhaustion at the final year of comprehensive school (Grade 9) were linked to a greater likelihood of later use of antidepressants after comprehensive school at the age of 16–20 years old. In line with this finding, students' higher levels of symptoms of school burnout in comprehensive school (exhaustion in Grades 7 and 9 and cynicism in Grade 9) were further found to be associated with students' self‐reported depressive symptoms after comprehensive schooling. The fact that the effect sizes (indicated by Nagelkerke's *R* ^2^ value and *R* ^2^ change value) were only moderate highlights that there are other variables not examined in the present study that also explain a notable proportion of the variation. Therefore, further studies need to examine risk factors beyond school burnout symptoms that contribute to the use of antidepressants and self‐reported depressive symptoms.

The results of the study, even while the effect sizes were moderate, support our hypothesis that school‐related problems can generalize outside the school context. The present findings are in line with previous research showing that the symptoms of school burnout may co‐occur or lead to more severe and all‐pervasive depressive symptoms (Fiorilli et al. 2017; Olivier et al. 2023; Salmela‐Aro, Savolainen, and Holopainen 2009). One possible explanation for these results can be derived from the demands–resources model (Salmela‐Aro et al. 2022; see also Bakker et al. 2003), which posits that burnout develops when demands (e.g., academic pressure and challenging tasks) exceed available resources (e.g., social support and motivational resources). When students face this imbalance, their energy may decrease, leading to exhaustion and other symptoms of school burnout. These symptoms, in turn, can increase vulnerability to more serious mental health conditions (Salmela‐Aro and Upadyaya 2014).

The study also provides new insights into the phenomenon by showing that symptoms of exhaustion are not only linked to depressive symptoms but also to a mental health condition that requires medical treatment. Although school burnout itself is not considered a medical condition (World Health Organization, n.d.), exhaustion symptoms seem to be linked to trajectories that can lead to further mental health problems. Sleep problems may be among the key mediating factors in this process, as they are linked to both symptoms of school burnout (Liu et al. 2021) and depression (Lovato and Gradisar 2014). It could be that students experiencing particularly exhaustion struggle with sleep, and due to prolonged problems with sleep, more all‐pervasive symptoms come to the fore.

In contrast, cynicism was related to the self‐reported depressive symptoms but not to the use of antidepressants. Thus, when a student loses a sense of meaning in school, this lack of meaning is likely to spill over into other life domains, leading to a broader loss of meaning in life (Wang et al. 2024), but this does not necessarily progress to clinical depression requiring medication. Our findings are consistent with the two‐process assumption of the demands–resources model (Demerouti et al. 2001). Exhaustion appears to reflect the energy‐depletion pathway, where sustained high demands erode personal resources and increase vulnerability to mental health problems that may require clinical intervention. Cynicism, in contrast, seems to align with the motivational pathway, contributing to depressive symptoms through disengagement and loss of meaning, but without necessarily progressing to clinical interventions. However, this hypothesis and other possible mechanisms should be examined in future studies.

In Finland, mental health problems are notable causes of sickness absences (Blomgren and Perhoniemi 2022), suggesting that mental health problems are not only a burden to individuals but also to society. In order to tackle this public health issue, action is clearly needed. Interventions should take place early on, as the present findings suggest that symptoms of school burnout present during the comprehensive school years may increase the risk of more severe mental health issues later. Although the effect sizes in our study were moderate, they appear to represent a meaningful and observable risk factor. In the context of population‐level prevention, these kinds of associations can justify early screening and targeted support, especially when the risk factor is identifiable within the school environment. The situation among students is concerning, and there has been an increasing trend in symptoms of school burnout, especially among girls (Read et al. 2022). Schools are therefore in a key position to recognize and address these symptoms to avoid them escalating into more serious mental health problems.

Symptoms of school burnout should not be viewed as the fault of individuals or signs of weakness, and attention should be drawn to the educational context and its practices that either promote or challenge students' mental health (Troy et al. 2022). Lowering the risk of burning out in daily school life could mean, for example, assigning a moderate number of tasks and tests, offering individual study arrangements, supporting habits that promote well‐being, and offering low‐threshold mental health services. Although specialized mental health care, such as psychiatric treatment, is sometimes necessary, shorter interventions conducted by school‐based professionals can be helpful for many (Parhiala et al. 2020). Additionally, it has been suggested that supporting students' coping strategies focused on seeking social support and problem solving could help reduce school burnout symptoms (Simonsen et al. 2025). Finally, when the focus is on prevention, early signs and possible risk factors for symptoms of school burnout (Vansoeterstede et al. 2023) should be observed and taken into consideration.

There are several suggestions and limitations for future research to attend to. First, although register‐based data provides information about participants' mental health problems while avoiding some of the biases linked to self‐reported questionnaires, it lacks information regarding specific diagnoses. Antidepressants can be prescribed for various reasons, including some non‐psychiatric conditions (Mercier et al. 2013). Thus, by including information on diagnoses (e.g., depression and anxiety), future research could complement and further validate the results of this study. Second, it should be considered that information regarding other kinds of treatments, such as psychotherapy, was not included. Moreover, some participants experiencing mental health problems might not have sought professional help in the form of medication. Therefore, the use of antidepressants cannot be considered a comprehensive indicator of the presence of mental health problems. Third, the present study did not examine the mechanisms behind the connection between the symptoms of school burnout and the use of antidepressants, which is an important topic for further investigations. Fourth, depressive symptoms at the comprehensive school were not controlled because they were not measured in our dataset. Although students with previous antidepressant usage were excluded in the present study, studies controlling previous depressive symptoms are also needed because it is possible that previous depressive symptomatology partly explains the associations found in the present study. Fifth, this study included only gender (and parental educational level in the additional analyses) as the controlled demographic factors. Thus, in future research, a broader set of demographic factors should be examined. Finally, our research findings demonstrated that school burnout symptoms in comprehensive school predicted subsequent start of medical treatment, that is, use of antidepressants, but it is possible that there is individual heterogeneity in the studied phenomenon. Thus, the future research line is to broaden the findings of the present study (found on average level) to examine the developmental trajectories of school burnout symptoms across school years and the associations of these possible different trajectories with the subsequent use of antidepressants.

## 5 Conclusion

The findings of the present study indicated that students' school‐context‐specific burnout symptoms during comprehensive school can lead to more severe mental health problems later in life. Overall, school burnout symptoms, exhaustion in particular, in comprehensive school should be taken seriously as they can increase the risk of both self‐reported depressive symptoms and clinically significant mental health conditions treated with medication. Providing support for students struggling with school burnout symptoms is important for the prevention of later age mental health problems.

Importantly, this study contributes to the existing literature by going beyond self‐reported measures and utilizing register‐based antidepressant data as a more objective indicator of clinically significant mental health outcomes. This approach strengthens the evidence for the long‐term consequences of school burnout and further highlights the need for early interventions to prevent negative outcomes later in life.

## Author Contributions

**Milja Parviainen** participated in the design of this study, performed the statistical analyses and interpretation of the data, and drafted the manuscript; **Kaisa Aunola** participated in the design of this study, provided guidance concerning the statistical analyses and interpretation of the data, and assisted with editing the manuscript; **Minna Torppa** participated in the design of this study, provided guidance concerning the interpretation of the data, and assisted with editing the manuscript; **Karoliina Koskenvuo** participated in the design of this study, provided guidance concerning register data and assisted editing the manuscript; **Miia Saarikallio‐Torp** participated in the design of this study, provided guidance concerning register data and assisted editing the manuscript; **Anna‐Maija Poikkeus** designed the First Steps follow‐up project of which this study is a part and assisted in editing the manuscript; **Kati Vasalampi** designed the School Path project of which this study is a part, participated in the design of this study, provided guidance concerning the interpretation of the data, and assisted with editing the manuscript. All authors read and approved the final manuscript.

## Funding

This study was supported by grants from the Academy of Finland (No. 268586, 299506, 276239, and 323773) and a grant from Ellen and Artturi Nyyssönen Foundation.

## Ethics Statement

The authors followed the ethical standards of the American Psychological Association when conducting the study.

## Conflicts of Interest

The authors declare no conflicts of interest.

## Data Availability

The data that support the findings of this study are available from the corresponding author upon reasonable request.

## Appendix A

### Measures

Parental educational level. Both parents of students were asked to report on their own and their spouse's highest levels of education. Information from both parents was combined to create a composite measure of total parental education for the family. In cases where data were available from only one parent, that value was used. Educational levels ranged from no post‐compulsory education to doctoral degrees, and their distribution closely aligned with the national educational distribution in Finland.

**TABLE A1:** Controlling for parental educational level in logistic regression analysis—Symptoms of school burnout at grades 6 (time 1), 7 (time 2), and 9 (time 3) as predictors of the use of antidepressants (0 = no use of antidepressants, 1 = use of antidepressants) at age 16–20.

	*β*	s.e.	sig.	OR	(95% CI)
STEP 1					
Gender	−0.600	0.349	0.085	0.549	(0.277–1.087)
Parental education	−0.054	0.108	0.619	0.948	(0.766–1.172)
STEP 2					
Gender	−0.463	0.383	0.226	0.629	(0.297–1.133)
Parental education	−0.043	0.110	0.698	0.958	(0.772–1.190)
Exhaustion/grade 6	0.210	0.229	0.360	1.233	(0.787–1.932)
Cynicism/grade 6	−0.101	0.228	0.656	0.904	(0.578–1.412)
Exhaustion/grade 7	−0.136	0.222	0.540	0.873	(0.564–1.349)
Cynicism/grade 7	0.146	0.240	0.544	1.157	(0.723–1.853)
Exhaustion/grade 9	0.405	0.193	0.036	1.499	(1.027–2.187)
Cynicism/grade 9	0.324	0.205	0.114	1.383	(0.925–2.067)

*Note:* The Nagelkerke *R* ^2^ for the final model was 0.10.

Abbreviations: 0, girl; 1, boy; 95% CI, 95% confidence interval; OR, odds ratio; s.e., standard error; *β*, standardized beta coefficient.

**TABLE A2:** Controlling for parental educational level in hierarchical regression analysis—School burnout symptoms at grades 6 (time 1), 7 (time 2), and 9 (time 3) as predictors of self‐reported depressive symptoms during the first year of upper secondary education (Time 4).

	STEP 1	STEP 2
	*β*	*β*
Gender	−0.207***	−0.162***
Parental education	0.007	0.014
Exhaustion/grade 6		0.065
Cynicism/grade 6		−0.008
Exhaustion/grade 7		0.164**
Cynicism/grade 7		−0.052
Exhaustion/grade 9		0.143**
Cynicism/grade 9		0.289***
	*F*(2, 454) = 10.044***	*F*(8, 448) = 18.023***
	*R* ^*2*^_change_ = 0.042***	*R* ^2^_change_ = 0.201***

Abbreviations: 0, girl; 1, boy; *β*, standardized beta coefficient.

** *p* < 0.01.

*** *p* < 0.001.

## References

[sjop70064-bib-0001] Bakker, A. B. , E. Demerouti , and W. B. Schaufeli . 2003. “Dual Processes at Work in a Call Centre: An Application of the Job Demands–Resources Model.” European Journal of Work and Organizational Psychology 12, no. 4: 393–417. 10.1080/13594320344000165.

[sjop70064-bib-0002] Bianchi, R. , J. Verkuilen , I. S. Schonfeld , et al. 2021. “Is Burnout a Depressive Condition? A 14‐Sample Meta‐Analytic and Bifactor Analytic Study.” Clinical Psychological Science 9, no. 4: 579–597. 10.1177/2167702620979597.

[sjop70064-bib-0003] Blomgren, J. , and R. Perhoniemi . 2022. “Increase in Sickness Absence due to Mental Disorders in Finland: Trends by Gender, Age and Diagnostic Group in 2005–2019.” Scandinavian Journal of Public Health 50, no. 3: 318–322. 10.1177/1403494821993705. 33615899 PMC9096587

[sjop70064-bib-0004] Demerouti, E. , A. B. Bakker , F. Nachreiner , and W. B. Schaufeli . 2001. “The Job Demands‐Resources Model of Burnout.” Journal of Applied Psychology 86: 499–512. 10.1037/0021-9010.86.3.499. 11419809

[sjop70064-bib-0005] Finnish Institute for Health and Welfare . 2024. “Kouluterveyskyselyn Aikasarjat Perusopetus 8. ja 9. lk, Lukio, Aol, 2006–2023 [Time Series From the School Health Promotion Study: Comprehensive School, Grades 8 and 9, Upper Secondary School, Vocational Education, 2006–2023].” https://sampo.thl.fi/pivot/prod/fi/ktk2/nuoret/fact_ktk2_nuoret?row=952513L&row=952810L&column=alue‐886778.&column=vuosi‐952479.&column=ka‐987089&column=taustatekija‐888288&column=sp‐888243&fo=1#.

[sjop70064-bib-0006] Fiorilli, C. , S. De Stasio , C. Di Chiacchio , A. Pepe , and K. Salmela‐Aro . 2017. “School Burnout, Depressive Symptoms and Engagement: Their Combined Effect on Student Achievement.” International Journal of Educational Research 84: 1–12. 10.1016/j.ijer.2017.04.001.

[sjop70064-bib-0007] Herrmann, J. , K. Koeppen , and U. Kessels . 2019. “Do Girls Take School Too Seriously? Investigating Gender Differences in School Burnout From a Self‐Worth Perspective.” Learning and Individual Differences 69: 150–161. 10.1016/j.lindif.2018.11.011.

[sjop70064-bib-0008] Jiang, S. , Q. Ren , C. Jiang , and L. Wang . 2021. “Academic Stress and Depression of Chinese Adolescents in Junior High Schools: Moderated Mediation Model of School Burnout and Self‐Esteem.” Journal of Affective Disorders 295: 384–389. 10.1016/j.jad.2021.08.085. 34492431

[sjop70064-bib-0009] Koutsimani, P. , A. Montgomery , and K. Georganta . 2019. “The Relationship Between Burnout, Depression, and Anxiety: A Systematic Review and Meta‐Analysis.” Frontiers in Psychology 10: 284. 10.3389/fpsyg.2019.00284. 30918490 PMC6424886

[sjop70064-bib-0010] Lee, M. Y. , and S. M. Lee . 2018. “The Effects of Psychological Maladjustments on Predicting Developmental Trajectories of Academic Burnout.” School Psychology International 39, no. 3: 217–233. 10.1177/0143034318766206.

[sjop70064-bib-0011] Leiter, M. P. , J. J. Hakanen , K. Ahola , S. Toppinen‐Tanner , A. Koskinen , and A. Väänänen . 2013. “Organizational Predictors and Health Consequences of Changes in Burnout: A 12‐Year Cohort Study.” Journal of Organizational Behavior 34: 959–973. 10.1002/job.1830.

[sjop70064-bib-0037] Lerkkanen, M.‐K. , P. Niemi , A.‐M. Poikkeus , E. Poskiparta , M. Siekkinen , and J.‐E. Nurmi . 2006–2016. The First Steps study [Alkuportaat]. University of Jyväskylä.

[sjop70064-bib-0012] Liu, X. , L. Zhang , G. Wu , R. Yang , and Y. Liang . 2021. “The Longitudinal Relationship Between Sleep Problems and School Burnout in Adolescents: A Cross‐Lagged Panel Analysis.” Journal of Adolescence 88: 14–24. 10.1016/j.adolescence.2021.02.001. 33588271

[sjop70064-bib-0013] Lovato, N. , and M. Gradisar . 2014. “A Meta‐Analysis and Model of the Relationship Between Sleep and Depression in Adolescents: Recommendations for Future Research and Clinical Practice.” Sleep Medicine Reviews 18, no. 6: 521–529. 10.1016/j.smrv.2014.03.006. 24857255

[sjop70064-bib-0014] Madsen, I. E. H. , T. Lange , M. Borritz , and R. Rugulies . 2015. “Burnout as a Risk Factor for Antidepressant Treatment—A Repeated Measures Time‐To‐Event Analysis of 2936 Danish Human Service Workers.” Journal of Psychiatric Research 65: 47–52. 10.1016/j.jpsychires.2015.04.004. 25943951

[sjop70064-bib-0015] Maslach, C. , W. B. Schaufeli , and M. P. Leiter . 2001. “Job Burnout.” Annual Review of Psychology 52: 397–422. 10.1146/annurev.psych.52.1.397. 11148311

[sjop70064-bib-0016] Mercier, A. , I. Auger‐Aubin , J.‐P. Lebeau , et al. 2013. “Evidence of Prescription of Antidepressants for Non‐Psychiatric Conditions in Primary Care: An Analysis of Guidelines and Systematic Reviews.” BMC Family Practice 14: 55. 10.1186/1471-2296-14-55. 23641784 PMC3648410

[sjop70064-bib-0017] Olivier, E. , A. J. S. Morin , V. Leo , and K. Salmela‐Aro . 2023. “The Interconnected Development of Depressive Symptoms and School Functioning From Mid‐Adolescence to Early Adulthood: A Piecewise Growth Mixture Analysis.” Journal of Educational Psychology 115, no. 3: 427–445. 10.1037/edu0000761.

[sjop70064-bib-0018] Parhiala, P. , K. Ranta , V. Gergov , et al. 2020. “Interpersonal Counseling in the Treatment of Adolescent Depression: A Randomized Controlled Effectiveness and Feasibility Study in School Health and Welfare Services.” School Mental Health 12: 265–283. 10.1007/s12310-019-09346-w.

[sjop70064-bib-0019] Parviainen, M. , K. Aunola , M. Torppa , M.‐K. Lerkkanen , A.‐M. Poikkeus , and K. Vasalampi . 2021. “Early Antecedents of School Burnout in Upper Secondary Education: A Five‐Year Longitudinal Study.” Journal of Youth and Adolescence 50: 231–245. 10.1007/s10964-020-01331-w. 33128134 PMC7875950

[sjop70064-bib-0020] Read, S. , L. Hietajärvi , and K. Salmela‐Aro . 2022. “School Burnout Trends and Sociodemographic Factors in Finland 2006–2019.” Social Psychiatry and Psychiatric Epidemiology 57: 1659–1669. 10.1007/s00127-022-02268-0. 35318486 PMC9288953

[sjop70064-bib-0021] Salmela‐Aro, K. , N. Kiuru , E. Leskinen , and J.‐E. Nurmi . 2009. “School Burnout Inventory (SBI): Reliability and Validity.” European Journal of Psychological Assessment 25, no. 1: 48–57. 10.1027/1015-5759.25.1.48.

[sjop70064-bib-0022] Salmela‐Aro, K. , J. Muotka , K. Alho , K. Hakkarainen , and K. Lonka . 2016. “School Burnout and Engagement Profiles Among Digital Natives in Finland: A Person‐Oriented Approach.” European Journal of Developmental Psychology 13, no. 6: 704–718. 10.1080/17405629.2015.1107542.

[sjop70064-bib-0023] Salmela‐Aro, K. , H. Savolainen , and L. Holopainen . 2009. “Depressive Symptoms and School Burnout During Adolescence: Evidence From Two Cross‐Lagged Longitudinal Studies.” Journal of Youth and Adolescence 38, no. 10: 1316–1327. 10.1007/s10964-008-9334-3. 19779808

[sjop70064-bib-0024] Salmela‐Aro, K. , X. Tang , and K. Upadyaya . 2022. “Study Demands‐Resources Model of Student Engagement and Burnout.” In Handbook of Research on Student Engagement, edited by A. L. Reschly and S. L. Christenson , 2nd ed., 77–93. Springer. 10.1007/978-3-031-07853-8_4.

[sjop70064-bib-0025] Salmela‐Aro, K. , and L. Tynkkynen . 2012. “Gendered Pathways in School Burnout Among Adolescents.” Journal of Adolescence 35, no. 4: 929–939. 10.1016/j.adolescence.2012.01.001. 22300678

[sjop70064-bib-0026] Salmela‐Aro, K. , and K. Upadyaya . 2014. “School Burnout and Engagement in the Context of Demands–Resources Model.” British Journal of Educational Psychology 84: 137–151. 10.1111/bjep.12018. 24547758

[sjop70064-bib-0027] Salokangas, R. K. R. , O. Poutanen , and E. Stengård . 1995. “Screening for Depression in Primary Care. Development and Validation of the Depression Scale, a Screening Instrument for Depression.” Acta Psychiatrica Scandinavica 92, no. 1: 10–16. 10.1111/j.1600-0447.1995.tb09536.x. 7572242

[sjop70064-bib-0028] Shorey, S. , E. D. Ng , and C. H. J. Wong . 2022. “Global Prevalence of Depression and Elevated Depressive Symptoms Among Adolescents: A Systematic Review and Meta‐Analysis.” British Journal of Clinical Psychology 61, no. 2: 287–305. 10.1111/bjc.12333. 34569066

[sjop70064-bib-0029] Simonsen, J. , M. Karrasch , M. Laine , and Å. Fagerlund . 2025. “Protective Factors Against School Burnout Symptoms in Finnish Adolescents.” Nordic Psychology 77, no. 1: 3–25. 10.1080/19012276.2023.2244678.

[sjop70064-bib-0030] Söderholm, F. , J. Viljaranta , R. Hirvonen , H. Tuominen , K. Lappalainen , and L. Holopainen . 2025. “The Development of Exhaustion, Cynicism, and Inadequacy in General Upper Secondary Education: Perceived and Received Support, Student Engagement, and Gender as Predictors.” Scandinavian Journal of Educational Research 69, no. 6: 1271–1287. 10.1080/00313831.2024.2419066.

[sjop70064-bib-0031] Tomaszek, K. , A. Muchacka‐Cymerman , A. Aypay , and F. Altınsoy . 2024. “Who Can Make Burned‐Out Students Feel Better and Self‐Efficient? Latent Profiles of Student Burnout and Its Association to Personal and Social Resources Among Polish and Turkish Early Adolescents.” Child Indicators Research 17: 2481–2502. 10.1007/s12187-024-10169-8.

[sjop70064-bib-0032] Troy, D. , J. Anderson , P. E. Jessiman , et al. 2022. “What Is the Impact of Structural and Cultural Factors and Interventions Within Educational Settings on Promoting Positive Mental Health and Preventing Poor Mental Health: A Systematic Review.” BMC Public Health 22: 524. 10.1186/s12889-022-12894-7. 35300632 PMC8927746

[sjop70064-bib-0033] Vansoeterstede, A. , E. Cappe , J. Lichtlé , and E. Boujut . 2023. “A Systematic Review of Longitudinal Changes in School Burnout Among Adolescents: Trajectories, Predictors, and Outcomes.” Journal of Adolescence 95, no. 2: 224–247. 10.1002/jad.12121. 36385709

[sjop70064-bib-0038] Vasalampi, K. , and K. Aunola . 2016–2023. The School Path: From First Steps to Secondary and Higher Education study [Koulupolku: Alkuportailta jatko‐opintoihin]. University of Jyväskylä.

[sjop70064-bib-0034] Wang, X. , M. Yang , L. Ren , et al. 2024. “Burnout and Depression in College Students.” Psychiatry Research 335: 115828. 10.1016/j.psychres.2024.115828. 38518519

[sjop70064-bib-0035] WHO Collaborating Centre for Drug Statistics Methodology . 2025. “International Language for Drug Utilization Research.” https://atcddd.fhi.no/.

[sjop70064-bib-0036] World Health Organization . n.d. “International Classification of Diseases 11th Revision (ICD‐11).” https://icd.who.int/en.

